# UK Doubles Its “World-Leading” Research in Life Sciences and Medicine in Six Years: Testing the Claim?

**DOI:** 10.1371/journal.pone.0132990

**Published:** 2015-07-23

**Authors:** Steven Wooding, Thed N. Van Leeuwen, Sarah Parks, Shitij Kapur, Jonathan Grant

**Affiliations:** 1 RAND Europe, Cambridge, United Kingdom; 2 Leiden University, Center for Science & Technology Studies (CWTS), Leiden, Netherlands; 3 King’s College London, Institute of Psychiatry, Psychology and Neuroscience, London, United Kingdom; 4 King’s College London, Policy Institute, London, United Kingdom; Max Planck Society, GERMANY

## Abstract

**Background:**

The UK, like some other countries, carries out a periodic review of research quality in universities and the most recent Research Excellence Framework (REF) reported a doubling (103% increase) in its “world leading” or so-called “4*” research outputs in the areas of life sciences and medicine between 2008 and 2014. This is a remarkable improvement in six years and if validated internationally could have profound implications for health sciences.

**Methods:**

We compared the reported changes in 4* quality to bibliometric measures of quality for the 56,639 articles submitted to the RAE 2008 and the 50,044 articles submitted to the REF 2014 to Panel A, which assesses the life sciences, including medicine.

**Findings:**

UK research submitted to the RAE and REF was of better quality than worldwide research on average. While we found evidence for some increase in the quality of top UK research articles, a 10-25% increase in the top 10%ile papers, depending upon the metrics used, we could not find evidence to support a 103% increase in quality. Instead we found that as compared to the RAE, the REF results implied a lower citation %ile threshold for declaring a 4*.

**Interpretation:**

There is a wide discrepancy between bibliometric indices and peer-review panel judgements between the RAE 2008 and REF 2014. It is possible that the changes in the funding regime between 2008 and 2014 that significantly increased the financial premium for 4* articles may have influenced research quality evaluation. For the advancement of science and health, evaluation of research quality requires consistency and validity – the discrepancy noted here calls for a closer examination of mass peer-review methods like the REF.

## Introduction

The UK was the first country to introduce a national framework for evaluating the research output of universities when it introduced the Research Assessment Exercise in 1986. However such systems are becoming increasingly relevant internationally. A review of such systems in 2010 identified 14 countries with national systems that evaluate research output ex post and where funding does, or soon will, be determined by this assessment [1]. Most of these systems emphasise an assessment of the ‘excellence’ of research, although they take different approaches to this assessment [2]. There are two principle approaches to assessment: peer review which is used in Spain, New Zealand, UK, Italy and Portugal and bibliometric approaches which are used in Norway, Denmark, Spain, Sweden, New Zealand and Belgium. The sophistication of these bibliometric approaches varies and is evolving from simple publication counts, to systems that reward publication in certain journals, or examine citation figures. In Australia each field of study can opt for either bibliometric or peer review based assessment methods. In the Netherlands a system combining peer review and advanced bibliometrics has been used since the 1990’s, with metrics only applied in those domains in which bibliometrics have any meaning with respect to communication cultures in the respective domains [3]. A major important difference between for example the UK and Netherlands situation is the absence of a linking between research assessment outcomes and funding in the latter country.

In the most recent exercise in in the UK in 2014, the Research Excellence Framework (REF) UK universities had to submit four “research outputs”, usually peer-reviewed articles, for each participating academic faculty member [4]. 154 UK universities submitted 191,150 research outputs from 52,061 academics [5]. Each of these outputs was evaluated by a panel of peer-reviewers who provided it with a score of 4* (world-leading), 3* (internationally excellent), 2* (recognised internationally) or 1* (recognised nationally). The most striking results were from Panel A (which covered the life sciences, including medical and allied health professions research) which reported an increase in the proportion of world-leading (4*) research from 11.8% [6] in the previous Research Assessment Exercise (RAE) in 2008 to nearly 23.9% in REF 2014 [7]. The purpose of this article is to test this claim against international independent measures of quality using bibliometric indicators.

Bibliometrics is the quantitative analysis of scientific publications and their citations, and in a research assessment context has a number of known advantages and disadvantages [8]. Citations imply the “use” of the article by peers in the field–and as such are an indirect measure of quality. In contrast to peer-review, bibliometric data are easily compiled, international comparisons are relatively easy to make, and most advanced bibliometric techniques contain a form of normalization for the field and year of publication–thus providing a rather fine-grained comparison [9]. On the other hand, bibliometrics is mostly relevant for journal articles and less so for books and book chapters [10], and sometimes papers can receive high citations precisely for publishing wrong or odd findings. However, on balance, especially when judging a large body of contributions, bibliometrics can provide an external indicator of research quality. Whereas bibliometrics is a ‘wisdom of the crowd’ approach that assumes citations equate to quality, peer review asks particular individuals to make particular judgements on research quality, often against particular criteria. [11] There is a long history of criticism of peer review, but it is often accepted as ‘a system full of problems but the least worst we have’ [12]. More recently a study have shown that the inter-rater agreement of peer reviewers for journal articles is as low as 0.23 [13]. Indeed, the use of bibliometrics in the UK RAE and REF have been a matter of debate [14] and while the REF was open to the use of bibliometrics, each Panel used them to varying degrees. Panel A guidance suggested that outcomes should be “informed” by citation metrics but REF evaluators were provided no standardised guidance as to how it was to be related to the rating of 3* or 4* [2].

To test whether the reported doubling of world-leading research quality in the REF was supported by bibliometric quality, we obtained details of all Panel A outputs submitted to the RAE 2008 and REF 2014, and compared them to various bibliometric indices, along with international performance on the same indicators in that time frame.

## Methods

We obtained all the outputs submitted for Panel A and B for both RAE and REF from the public website [15]. The outputs submitted for REF were categorised into journal articles, chapters, books and other kinds of research communications. Since citation metrics are most relevant for journal articles, we restricted further evaluation to these research outputs which formed 97%/99% of the total outputs in Panel A for RAE/REF. The RAE and REF changed the nomenclature of panels and subpanels and to ensure comparability, we transformed all the RAE panel data into REF panel terminology, thus allowing a direct comparison.

The RAE/REF articles were matched against the Centre for Science and Technology Studies, Leiden (CWTS) version of the Web of Science database–which contains information on over 42 million articles from over 18 thousand journals and tracks more than 555 million citations [16]. To compare like-with-like, the citation to a particular article was compared to all other articles in the same field and from the same year of publication–allowing the determination of the worldwide “percentile” of that article. The database allocates all articles into scientific fields, some 250 Journal Subject Categories, of which about 80 relate directly to Medical and Life Sciences. Some articles are in journals that related to more than one category, in which case the overall standing of the article is the sum of its fractional standing in the fields to which it is assigned. We excluded self-citations in our analysis as we wanted to measure externally received citation impact, thereby excluding up-front any source of distortion. Finally, to validate our use of bibliometrics we examined how commonly the journal articles in our RAE/REF set referred to other articles which were also in this dataset, a measure called ‘internal coverage’, figures higher than 70% provide valid and stable measures of bibliometric indicators [17].

The CWTS database also allows access to all other (not REF submitted) articles published by UK authors within that timeframe. Since the fields of Medical and Life Sciences in this database are largely coterminous with Panel A in RAE/REF, we obtained bibliometric data regarding UK vs. World papers 2001–2013 from these fields of the database.

With this data available for all RAE and REF journal articles we asked the following questions: a) how many RAE/REF articles were available for bibliometric analysis?; b) how did the quality of articles submitted in the RAE vs. REF compare in terms of international percentile standing; c) how did quality of all UK Medical and Life Sciences articles (whether submitted to RAE/REF or not) change during that period?; d) has there been a lowering of the percentile thresholds at which papers are labelled 3*/4*?; and e) is this change of threshold seen only in Panel A (Life sciences) or also in Panel B (Engineering and physical sciences).

## Results

### Details of articles submitted for RAE and REF that were accessible

The REF Panel A received 50,044 articles, reflecting a fall in overall number from 56,639 received in RAE 2008. Of these, 96% of the RAE and 98% of the REF articles were linked to the CWTS Web of Science database and thus amenable to bibliometric analyses. RAE and REF rules allow the same paper to be submitted to more than one UoA, and from more than one institution, the number of duplicate papers identified was 12% for the RAE and 14% for the REF. We have analysed unique papers submitted as a better measure of overall quality of UK research. Finally–as is standard practice in bibliometric analyses—we excluded papers that were not articles or reviews, and weighted letters as 0.25 of a paper. This excluded a further 1% of RAE papers and 2% of REF papers. The measure of ‘internal coverage’ for these articles was 85% and 87% respectively–suggesting that the access, linking and citation patterns for the RAE and REF for Panel A are comparable.

### There has been an improvement in bibliometric quality of the UK’s research

**Fig 1:**
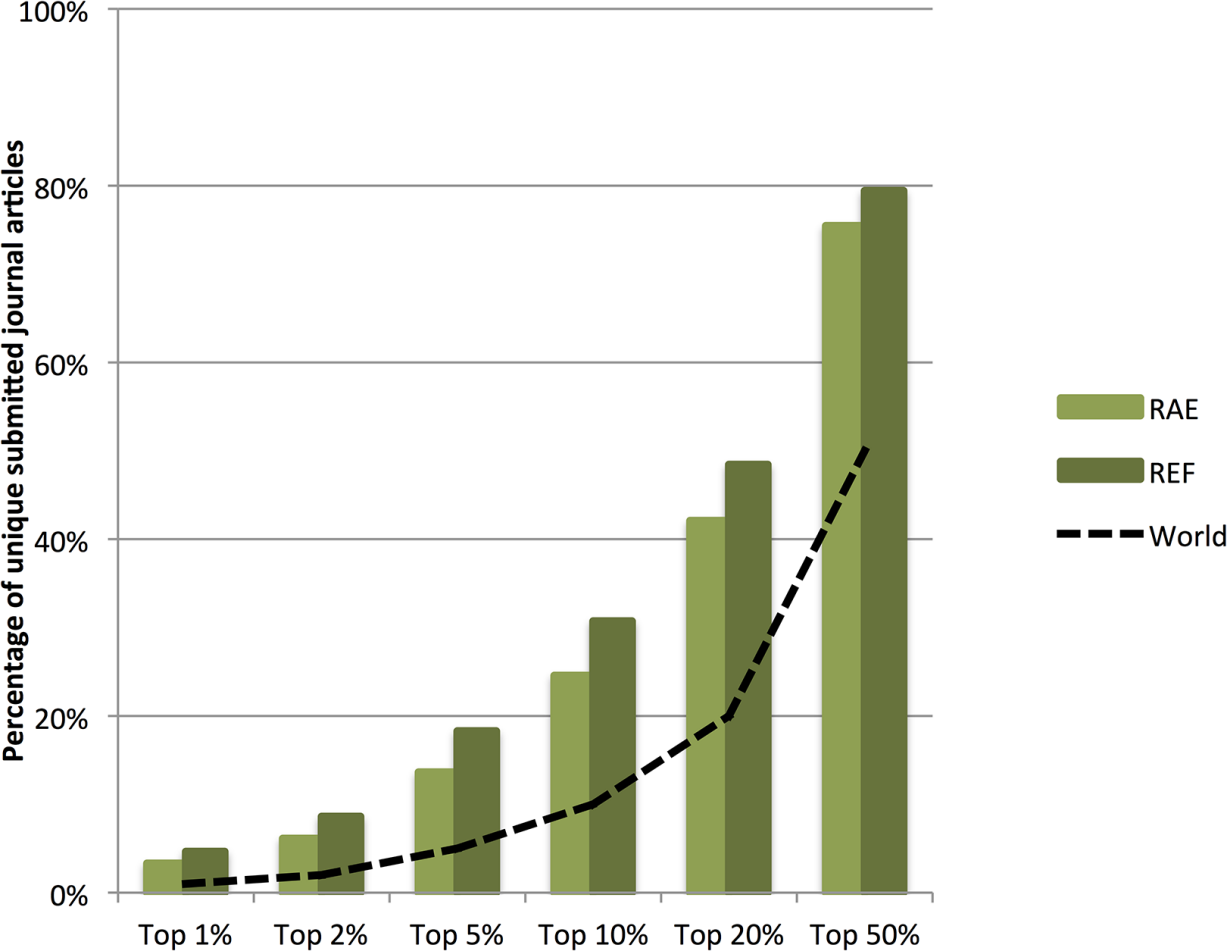
Percentage of the total journal articles submitted in RAE and REF to Panel A that were in the respective percentiles of their field. The dashed-line represents the world-average for the field i.e. 1% of papers in top 1% etc.

We examined the fraction of submitted journal articles that exceed a series of worldwide percentile thresholds, identifying those papers that fell into the top 1%ile through to the top 50%ile. Fig 1 shows the extent to which submitted research in the RAE and REF exceeded the world average. While both the RAE and REF exceeded the worldwide average, the REF submitted research was higher than the RAE at all top percentile levels, showing a nearly 25% improved performance at the 10%ile level.

**Fig 2:**
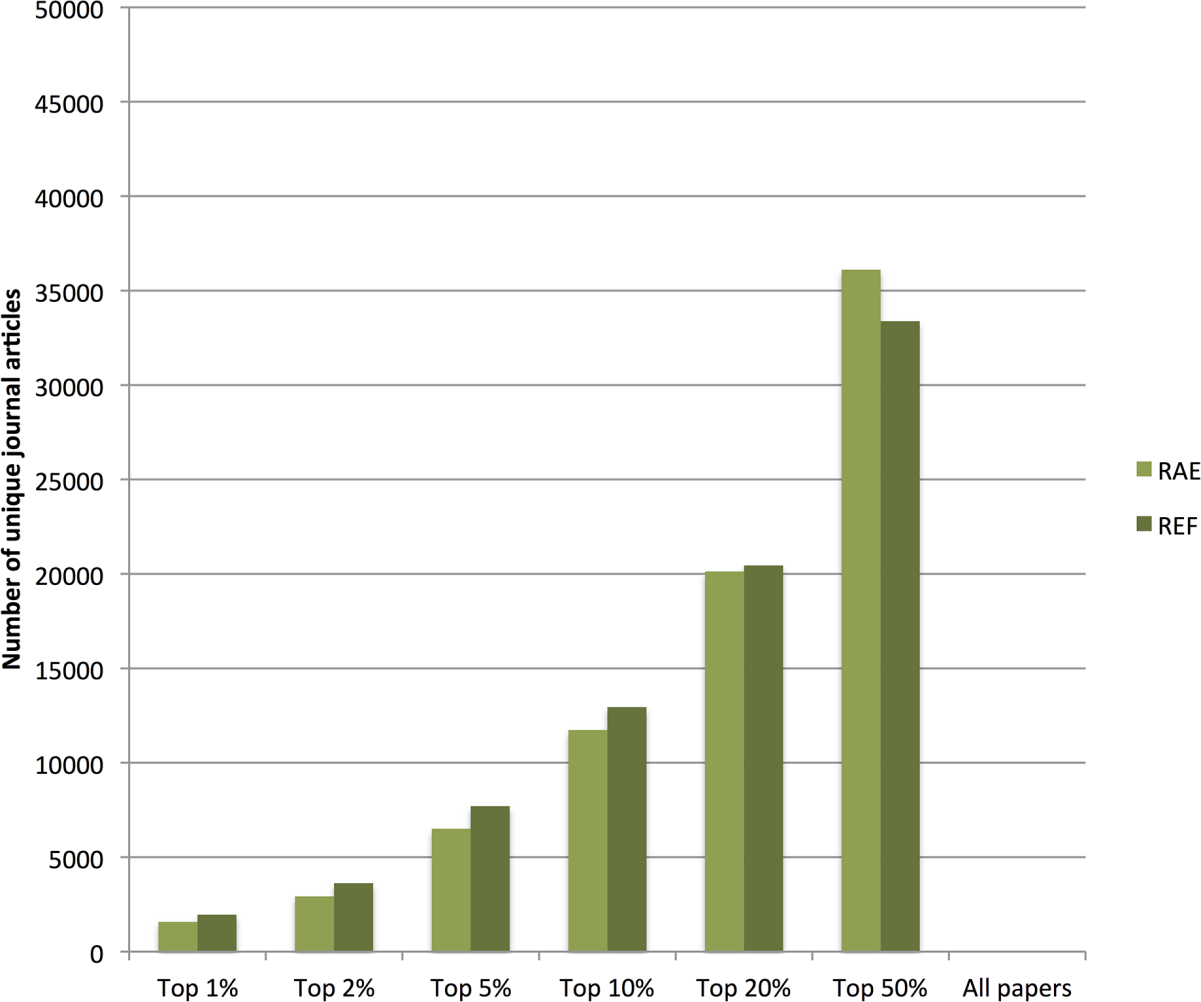
Total number of journal articles submitted in RAE and REF to Panel A that were in the respective percentiles of their field.

Since both the RAE and REF were selective exercises and universities were at liberty to submit their best researchers and outputs—this increase in relative quality could be due to increased selectivity of submission as a total of 9% fewer articles were submitted in the REF than in the RAE. Fig 2 displays the absolute number of articles, submitted in the RAE and REF and their percentile ratings in their respective fields. The absolute number of journal articles in the highest percentiles were still higher in REF versus the RAE–registering a 10% absolute increase in top 10%ile papers.

**Fig 3:**
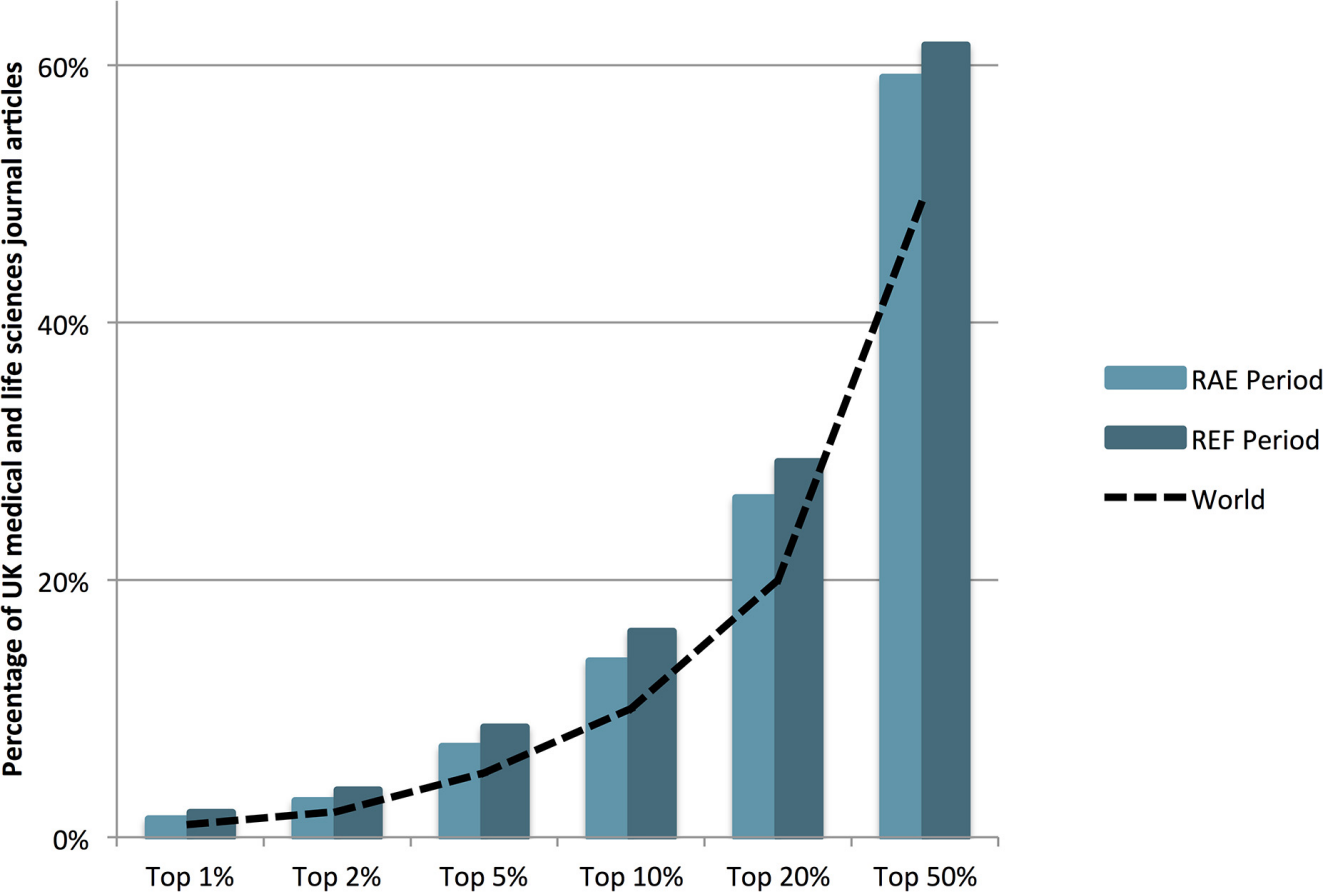
The percentage of journal articles published in the UK in the Medical and Life Sciences in the RAE and REF submission periods that were in the respective percentiles of their field. The dashed-line represents the world-average for the field i.e. 1% of papers in top 1% etc.

Panel A in RAE/REF assessed some fifty thousand articles, but this accounts for only a sixth of the overall research output of the UK in the domain of Medical and Life Sciences. To get a complete view of the change in quality and quantity, we compared the change in total volume and quality of the UK outputs, as compared to the world, in the RAE and REF periods. During the RAE period the UK published 288,327 journal articles in Medical and Life Sciences against a world total of 3,311,114, accounting for 8.7% of the world output. In the REF period, the UK produced 299,628 journal articles against a world output of 3,870,031, thus accounting for a smaller 7.7%, of the overall world output. The percentage of these papers that were in the different percentile bands is shown in Fig 3. UK’s contribution to the top 10%ile papers increased by 17% during this period.

### The citation equivalent of 4* and 3* has fallen between RAE 2008 and REF 2014

**Fig 4:**
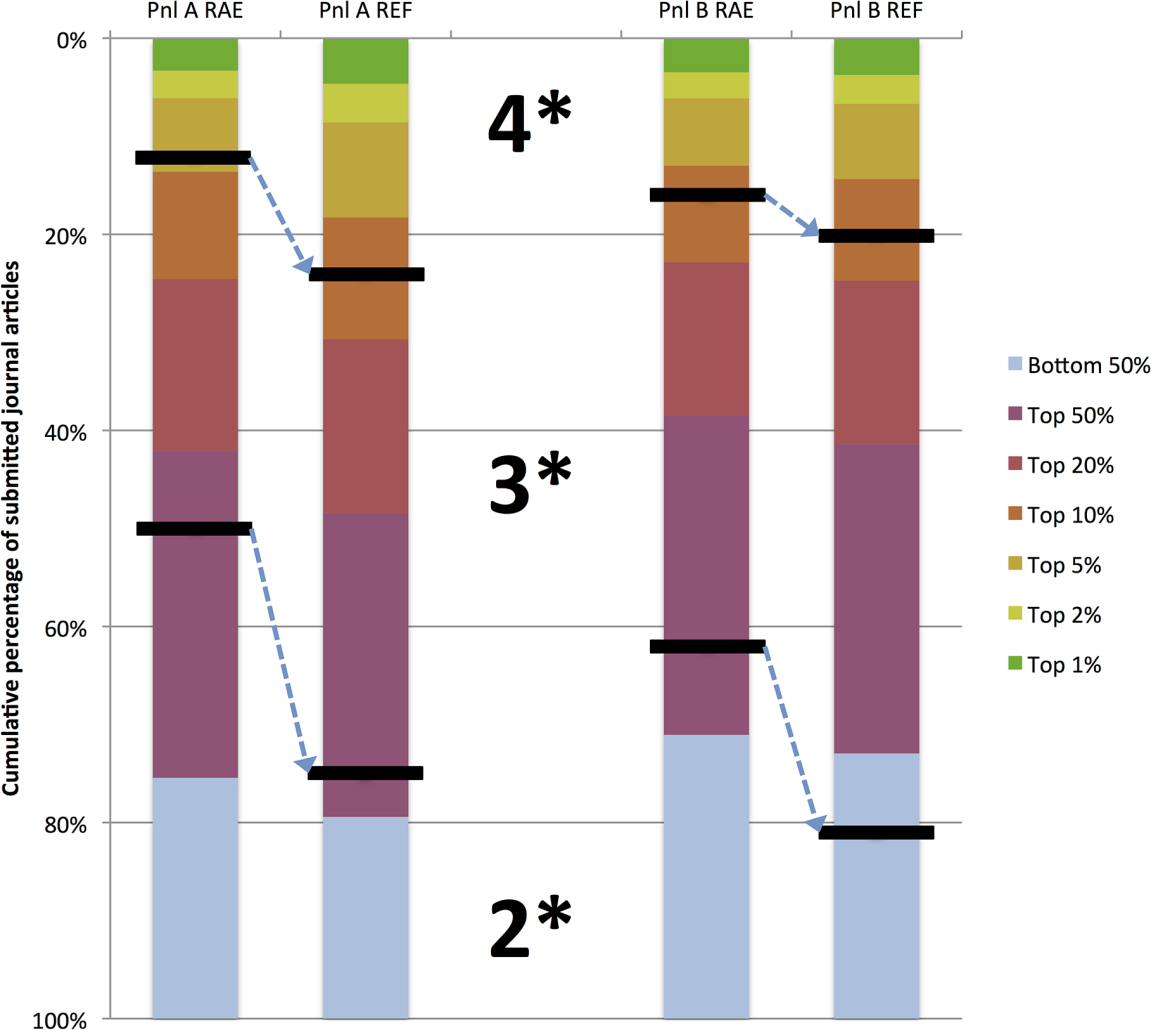
The stacked bars show the percent of the articles in the different percentiles worldwide in Panel A (Pnl A) and Panel B (Pnl B—Engineering and physical sciences) in the RAE and REF. The horizontal line through the stack shows the threshold equivalent which would give the same 4*/3* outcomes that were reported in RAE and REF. The dashed arrows shows the downward shift in threshold as compared to bibliometric performance.

While the REF provides a list of all the papers submitted, it does not provide the individual ratings. Nonetheless, one can relate the percentage of 4*/3*/2* journal articles in REF to their bibliometric qualities in aggregate. Using linear interpolation, in the RAE 2008, as many articles were classified as 4* as were in the top 4.4%ile of world outputs. In the REF 2014, this threshold dropped to 7.3%ile of world outputs. The lowering of other thresholds is displayed in Fig 4.

To examine if this was a phenomenon selective to Panel A, or seen within other panels where bibliometric analysis may be relevant, the results of a similar analysis of Panel B data are shown in Fig 4. The data shows a decrease in threshold between RAE and REF in Panel B as well–though the relative decrease appears less severe.

## Discussion

The availability of all the submitted research outputs for RAE and REF, and their cross-linkage to the CWTS Web of Science database made it possible to assess them for their bibliometric qualities. The bibliometric evidence supports some increase in the quality of UK research in these domains in the REF period versus the RAE period. However, there is a remarkable disparity between the level of improvement indicated by bibliometric indices (between 10 to 25% depending on indicator) and panel-rated improvement of 103% in “world leading” (4*) outputs. This difference bears closer exploration.

When the results of the REF were released other commentators were also struck by this increase in quality. One of the explanations proposed was that the enhanced research spend in the area of health sciences, some £6 billion by the National Institute of Health Research (NIHR) since 2006 which has gone selectively to medical sciences, may account for this remarkable increase in quality [18]. However, such an explanation is not supported by the fact that some sub-panels within Panel A which receive no funding from NIHR (e.g. Biological Sciences, or Food and Veterinary Sciences) showed an even higher increase in self-rated world-leading 4* quality (129% and 165% respectively) suggesting that the two are likely unrelated.

It is important to recognize that in overall research output, the share of the UK in the domain of Medical and Life Sciences has actually fallen–from 8.7% to 7.7%, not due to a decrease in absolute number of articles, but, due to a faster increase in the other publishing nations of the world. Despite this the UK seems to have held its own in terms of world-leading outputs, nominally the top 10%ile, in both relative and absolute numbers. But, none of these findings are compatible with the nearly “doubling” of world-leading quality reported in the REF. Thus, at least when compared to bibliometric indicators, the most likely explanation is that the REF Panel A used a somewhat lower threshold of acceptance for a 4* level, as compared to the RAE panels.

### Shift in relative threshold

REF ratings have very important meaning in UK academic circles as they are more than just an academic exercise in peer-review–the distribution of £1.6 billion per annum hinges on these results. While the results have no implications for a given individual, the REF results have huge implications for the relative standing of fields, the research funding of universities, and funding allocated within universities to different research groups. It is understandable then that the REF evaluations are likely to be influenced by these external factors. When the panelists were assessing RAE 2008, the prevailing funding formula was such that 4*, 3* and 2* outputs would be rewarded financially in a ratio of 7:3:1. However, after RAE 2008 result came out, the rules were changed and funding for 2* was eliminated. Thus, as the REF panels were assessing papers they knew that 2* papers would draw no financial credit. It is interesting then that there was an increase in 4* across all the subpanels of Panel A (from an average of 11.8% to 23.9%) and an almost equivalent decrease in 2* (36% to 22%). This change in financial weightings is of course not only true of Panel A, but also Panel B, and not surprisingly Panel B also saw a similar shift to 4*–though to a lesser degree. And as Fig 4 shows, this shift was associated with a change in threshold as compared to bibliometric indicators.

It is important to qualify the limitations of this analysis. We have limited ourselves to journal article research outputs, as bibliometrics of book chapters and other forms of outputs are considered less valid. Insofar as 97–99% of all submissions to Panel A are journal articles across RAE and REF, and as we were able to access bibliometric data on 96%-98% of them–we feel that the few research outputs that have been missed cannot account for the main results. It is important to emphasize that RAE/REF databases do not reveal 4*/3* scorer of individual articles. Thus, our analysis does not in any way claim to question the individual judgement of quality–but only the overall conclusions of the exercise. Third, the RAE/REF evaluation entailed not only Panel A and B, but also Panels C (Social Sciences) and D (Arts and Humanities). It is generally acknowledged that citation analysis is less valid in these domains^12^ and therefore we have refrained from analysing them in this context. And finally, the REF exercise, like the RAE, evaluates not only research outputs but also their impact on society (for REF) and the vitality of the university research environments (for both exercises)–the bibliometric analysis only relates to the former and therefore these findings have no bearing on the panel judgements of the other areas (which are also reported using the same star rating).

To conclude, the recent REF results suggest a doubling in the “world leading” quality of UK life sciences. We do not find support for this claim in bibliometric indicators of papers submitted to the REF or in UK Medical and Life Sciences output more generally. It is plausible that changes in the financial consequences of the RAE vs. REF exercise may have influenced university submission behaviour or panel judgements. Without access to ratings of individual papers it is difficult to do more than to raise this possibility. Insofar as these REF ratings have implications for rankings of UK departments within a field, inter-field comparisons within the UK, and claims regarding the position of UK science in the context of worldwide output–the discrepancy we have highlighted is of concern. Bibliometrics are only one measure of scientific quality, and do not replace peer-review. However, when the two diverge rather markedly–it deserves comment and further attention.

### Research in context

#### Evidence before this study

This is a novel study that uses the results of RAE 2008 and the recently published REF 2014 results, and links this with existing bibliometric databases. As far as we are aware this is the first time someone has linked these data to test the validity of the peer review led assessment of research outputs through REF.

#### Added value of this study

The analysis adds to discussions about the future of REF, the use of metrics in the assessment of research quality and validity of peer review processes.

#### Implications of all available evidence

The REF results suggest a doubling in the “world leading” quality of UK life sciences. We do not find evidence to support this claim in bibliometric indicators of papers submitted to the REF or in UK Medical and Life Sciences output more generally.

## Supporting Information

S1 Table Underlying aggregate data for figures presented and cited in paper. (XLSX)
